# Investigating the zoonotic origin of the West African Ebola epidemic

**DOI:** 10.15252/emmm.201404792

**Published:** 2014-12-30

**Authors:** Almudena Marí Saéz, Sabrina Weiss, Kathrin Nowak, Vincent Lapeyre, Fee Zimmermann, Ariane Düx, Hjalmar S Kühl, Moussa Kaba, Sebastien Regnaut, Kevin Merkel, Andreas Sachse, Ulla Thiesen, Lili Villányi, Christophe Boesch, Piotr W Dabrowski, Aleksandar Radonić, Andreas Nitsche, Siv Aina J Leendertz, Stefan Petterson, Stephan Becker, Verena Krähling, Emmanuel Couacy-Hymann, Chantal Akoua-Koffi, Natalie Weber, Lars Schaade, Jakob Fahr, Matthias Borchert, Jan F Gogarten, Sébastien Calvignac-Spencer, Fabian H Leendertz

**Affiliations:** 1 Institute of Tropical Medicine and International Health, Charité – Universitätsmedizin BerlinBerlin, Germany; 2 Epidemiology of Highly Pathogenic Microorganisms, Robert Koch InstituteBerlin, Germany; 3 Centre for Biological Threats and Special Pathogens, Robert Koch InstituteBerlin, Germany; 4 Wild Chimpanzee Foundation, at Max Planck Institute for Evolutionary AnthropologyLeipzig, Germany; 5 German Centre for Integrative Biodiversity ResearchLeipzig, Germany; 6 Department of Primatology, Max Planck Institute for Evolutionary AnthropologyLeipzig, Germany; 7 Eidolon ekologiKållered, Sweden; 8 Institute of Virology, Philipps-University MarburgMarburg, Germany; 9 Laboratoire National d'Appui au Développement Agricole, Laboratoire Central de la Pathologie AnimalBingerville, Côte d'Ivoire; 10 Research Center for the Development and Teaching Hospital, Université Alassane Ouattara de BouakeBouake, Côte d'Ivoire; 11 Institute of Experimental Ecology, Ulm UniversityUlm, Germany; 12 Department of Migration and Immuno-Ecology, Vogelwarte Radolfzell, Max Planck Institute for OrnithologyRadolfzell, Germany; 13 Zoological Institute, TU BraunschweigBraunschweig, Germany; 14 Department of Biology, McGill UniversityMontreal, QC, Canada

**Keywords:** bat, Ebola, West Africa, wildlife, zoonosis

## Abstract

The severe Ebola virus disease epidemic occurring in West Africa stems from a single zoonotic transmission event to a 2-year-old boy in Meliandou, Guinea. We investigated the zoonotic origins of the epidemic using wildlife surveys, interviews, and molecular analyses of bat and environmental samples. We found no evidence for a concurrent outbreak in larger wildlife. Exposure to fruit bats is common in the region, but the index case may have been infected by playing in a hollow tree housing a colony of insectivorous free-tailed bats (*Mops condylurus*). Bats in this family have previously been discussed as potential sources for Ebola virus outbreaks, and experimental data have shown that this species can survive experimental infection. These analyses expand the range of possible Ebola virus sources to include insectivorous bats and reiterate the importance of broader sampling efforts for understanding Ebola virus ecology.

## Introduction

To date, five Ebola virus species have been described, with the highest case fatality rates caused by the *Zaire Ebola virus* (EBOV: ∼88%). Since its discovery in 1976, EBOV has been responsible for several outbreaks in Central Africa. Although outbreaks likely have a zoonotic origin, it has rarely been possible to formally link EBOV outbreaks to a given animal reservoir. Thus, both EBOV ecology and factors facilitating their zoonotic transmission remain largely unknown. Two main modes of transmission into human populations have been suggested: either direct contact to a reservoir or contact to other wildlife that also contracts EBOV from the reservoir. Non-human primates and duikers are susceptible hosts for EBOV, yet the high pathogenicity of EBOV in both captive and wild populations suggests they do not represent the reservoir (Olival & Hayman, 2014). In contrast, some fruit (*Epomophorus wahlbergi*) and insectivorous bats (*Chaerephon pumilus* and *Mops condylurus*) have been shown to survive experimental EBOV infections (Swanepoel *et al*, 1996). Hints of active infections in wild populations have been revealed by sporadic EBOV RNA detection in three fruit bat species (*Epomops franqueti*,*Hypsignathus monstrosus,* and *Myonycteris torquata*; Leroy *et al*, 2005) as well as antibody detection in those species and *Eidolon helvum*,*Epomophorus gambianus*,*Micropteropus pusillus, Mops condylurus, Rousettus aegyptiacus,* and *Rousettus leschenaultii* (Olival & Hayman, 2014). This suggests bats may be an EBOV reservoir. Further, direct infection by bats is plausible, given that bats, especially fruit bats, are frequently hunted and consumed as bushmeat (Mickleburgh *et al*, 2009).

**Figure 1:**
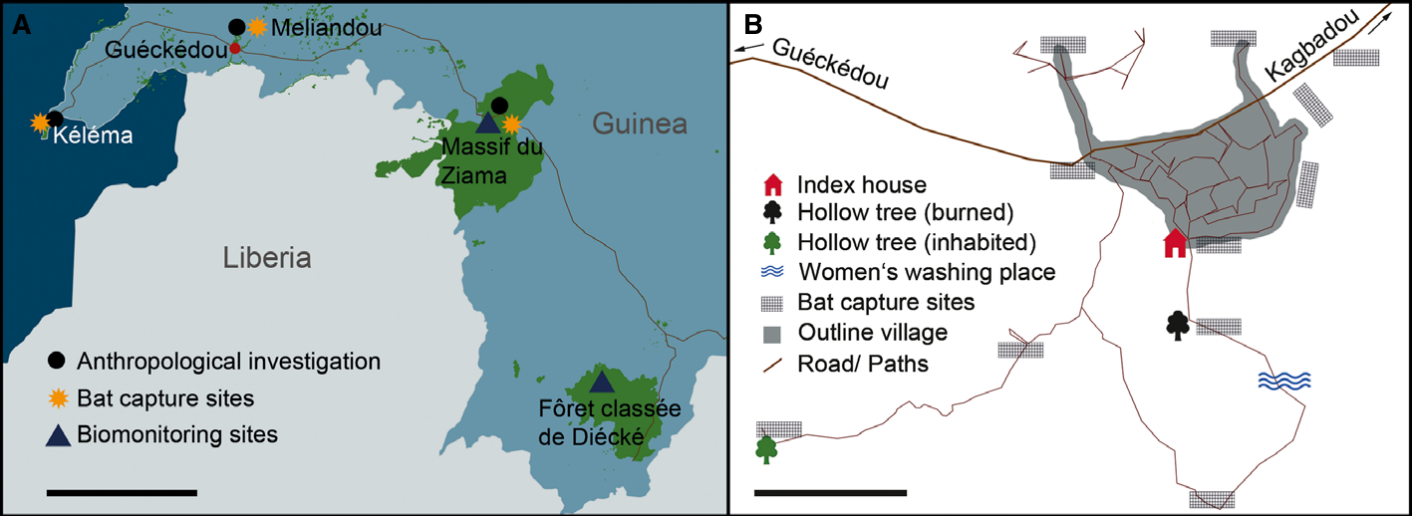
Sampling and investigation locations In southeastern Guinea (Sierra Leone, Guinea, and Liberia are visible); scale bar stands for 50 km.In and around the index village, Meliandou; scale bar stands for 100 m.

Since December 2013, a severe epidemic of Ebola virus disease (EVD) caused by EBOV has been occurring in West Africa (Baize *et al*, 2014). While there is some epidemiological evidence that a member of the *Ebola virus* genus may have circulated in Guinea before (Boiro *et al*, 1987; Schoepp *et al*, 2014), the current epidemic represents the first proven emergence of *Zaire Ebola virus* in West Africa (Calvignac-Spencer *et al*, 2014; Dudas & Rambaut, 2014). Epidemiological and genomic analyses suggest a single zoonotic transmission event followed by subsequent human-to-human transmission (Gire *et al*, 2014). The index case was a 2-year-old boy, who lived in Meliandou, a small village near Guéckédou in the Republic of Guinea (Supplementary Information, section A; Baize *et al*, 2014). We confirm the index case but present a slightly different timeline of subsequent transmission (Supplementary Information, section B). From there, the virus spread into other areas of Guinea and then Sierra Leone, Liberia, Nigeria, Senegal, USA, Spain, and Mali, representing the largest ever recorded outbreak with 17,145 cases and 6,070 deaths (as of December 3, 2014; World Health Organization, http://www.who.int/csr/disease/ebola/situation-reports/en/). We led a 4-week field mission in southeastern Guinea in April 2014, just after EBOV was confirmed as the cause of this epidemic, to examine human exposure to bats and other bushmeat, survey local wildlife in the last remaining forests of the area, and capture and sample bats in the index village as well as in neighboring forests (Fig1).

## Results

**Figure 2:**
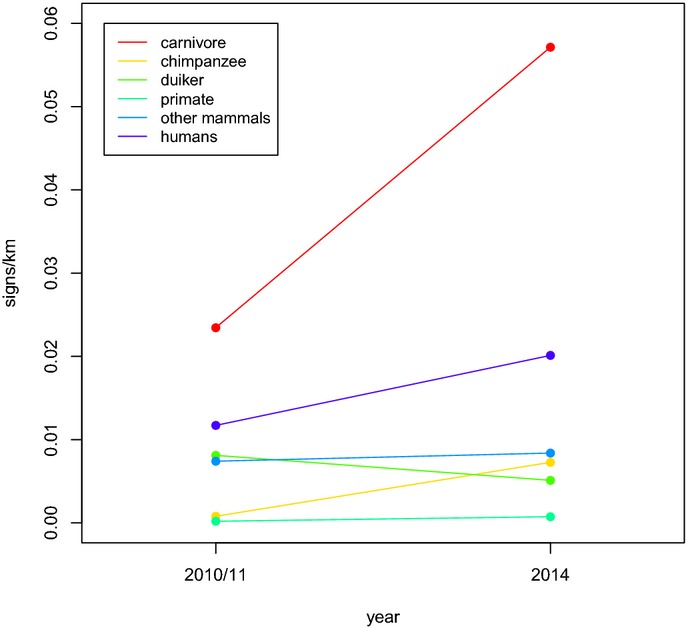
Wild animal and human densities in protected areas of southeastern Guinea before and after the onset of the EVD epidemic Signs per transect km were used as a proxy for target density.

Our surveys of the remaining large mammals in the only two protected areas in proximity to Meliandou (Fig1A) suggest they have not experienced a major decline; in fact, carnivore and chimpanzee (*Pan troglodytes verus*) populations may have increased (Fig2; Supplementary Table S1; Supplementary Information, section C). This contrasts with observations made during previous EBOV outbreaks, which reported a heavy toll on wild great apes (Walsh *et al*, 2003). Regional authorities, hunters, and women of the village stated that primates are rare in southeastern Guinea and that the few that remain are difficult to hunt. Most large game consumed in the region arrives smoked, from distant regions such as the Fouta Djallon in northwestern Guinea, and Liberia, making it an unlikely source of infection. Would contaminated fresh bushmeat have been brought to the village by a hunter, the latter would likely be among the first cases, as observed in several outbreaks in the Congo Basin (Georges *et al*, 1999; Khan *et al*, 1999; Leroy *et al*, 2004; World Health Organization, http://www.who.int/mediacentre/news/ebola/2-september-2014/en/). However, only children and women presented symptoms or died in the beginning of the current epidemic, and the sole survivor of the index case's family is the father, who had not lived in household for several years and was reported never to have been a hunter. Collectively, this suggests that larger wildlife did not serve as an intermediate amplifier leading to the infection of the index case.

In contrast, bat hunting was commonly described in the region. Men of Meliandou and six other neighboring villages reported opportunistically hunting fruit bats throughout the year (Supplementary Fig S1; Supplementary Information, section B). Officials and hunters interviewed in Meliandou and Guéckédou mentioned very large fruit bat colonies, but only in two relatively distant regions of southeastern Guinea; yearly, bat migrations were reported to bring large numbers of fruit bats to the region of Kéléma, while caves housing large colonies were reported to exist in the Ziama Biosphere Reserve (Fig1A). Insectivorous bats were reported to be commonly found under the roofs of houses and similar hides in the villages. These bats are reportedly targeted by children, who regularly hunt and grill them over small fires (Supplementary Information, section B).

During our field investigations in southeastern Guinea, we captured bats in the regions of Meliandou (*N *= 88 in Meliandou itself and *N *= 20 in Kagbadou), Kéléma (*N *= 4), and Ziama (*N *= 57). We could assign bats to a minimum of 13 species representing six families (Supplementary Table S2; Supplementary Information, section D). Three of the species captured—*Eidolon helvum*,*Hypsignathus monstrosus,* and *Mops condylurus*—were previously reported as Ebola virus positive (either through PCR or serologically; Olival & Hayman, 2014). No EBOV RNA was detected in any of the PCR-tested bat samples. Attempts to demonstrate the presence of IgG antibodies against Ebola viruses were inconclusive (data not shown). Further serological investigations to determine whether these bats were exposed to an Ebola virus will be required.

**Figure 3:**
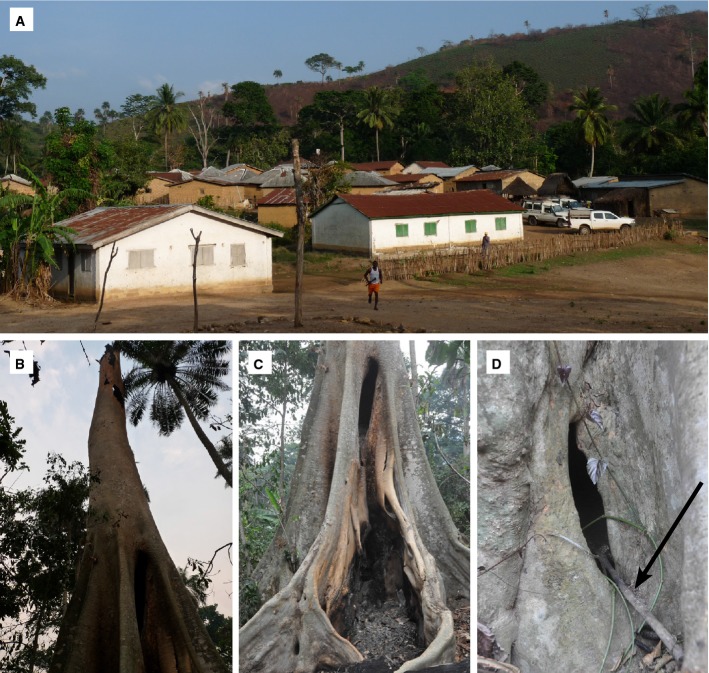
Meliandou and the burnt tree that housed a bat colony A The village of Meliandou.B–D The burnt hollow tree; in (D), the arrow points at a stick, most probably left there by children.

To understand how the 2-year-old boy may have contracted EBOV, we conducted detailed observations in Meliandou over 8 days. Meliandou is a small village of 31 houses, surrounded by farmland and few larger trees (Fig3A). We found a large tree stump situated approximately 50 m from the home of the index case near an often-used path leading to a small river used by women for washing (Fig1B). Villagers reported that children used to play frequently in this hollow tree (Fig3B–D). When we arrived, the tree had been mostly burned and only the stump and fallen branches remained (Fig3C and D). Villagers reported that it burned on March 24, 2014 and that once the tree caught fire, a “rain of bats” started and a large number of bats were collected for consumption (see Materials and Methods). The bats were described as *lolibelo*, that is, small, smelly bats with a long tail. We found no evidence of additional zoonotic transmission events stemming from the consumption of these bats, but villagers reported disposing of them after a ban on bushmeat consumption was announced the following day. Deep sequencing of a short 16S mitochondrial DNA fragment amplified from ash and soil samples collected within and around this burned tree revealed that five of 11 samples contained sequences that could be assigned to *Mops condylurus* (100% identity; Supplementary Fig S2, Supplementary Information, section E). This species matches the description provided by villagers.

## Discussion

Collectively, our results allow us to exclude the hypothesis that a major die-off in wildlife led to the human epidemic and to pinpoint two hypotheses implicating bats:

### H1

Fruit bats, the commonly suspected EBOV reservoir, are hunted for their meat in the region. We captured fruit bats directly in Meliandou, suggesting transmission from a fruit bat reservoir may have been possible (Leroy *et al*, 2005).

### H2

Consumption of fruit bats in the household is an unlikely source of infection for the index case; no hunters were members of this household and a food item-borne transmission would likely have affected adults before or concurrently with the index case. Under the assumption that the 2-year-old boy was indeed the index case, a source of infection unrelated to food items consumed in the home might be more plausible. The close proximity of a hollow tree housing a large colony of free-tailed bats (i.e., insectivorous bats), of a species for which serological evidence also suggests EBOV exposure (Pourrut *et al*, 2009), provided opportunity for infection. Children regularly caught and played with bats in this tree. Free-tailed bats were suggested as a source for the first *Sudan Ebola virus* outbreaks (World Health Organization/International Study Team, 1978). Risk of infection via exposure to high-density bat colonies is well documented for the Marburg virus (MARV), a virus also in the *Filoviridae* family (Amman *et al*, 2012).

Our findings support the idea that bats were the source of the current EVD epidemic in West Africa and enlarge the list of plausible reservoirs to include insectivorous bats. Future sampling campaigns, in-depth serological studies, and modeling efforts should take into account the possibility that fruit bats may not always be the ultimate source of EVD outbreaks.

We note that culling or eviction attempts that targeted bat colonies to mitigate human–wildlife conflicts and reduce disease transmission were frequently unsuccessful; in some cases, even producing effects opposite to those desired by the initiative (Amman *et al*, 2014). Health education initiatives should inform the public about the disease risk posed by bats, but also that these animals perform crucial ecosystem services with direct and invaluable benefits to humans, while providing methods for minimizing contact with bats. Similarly, while the village of Meliandou had the misfortune to be where the zoonotic transmission event occurred, care needs to be taken to avoid retribution attacks and stigmatization of the region.

## Materials and Methods

We established a multidisciplinary field team, consisting of an anthropologist to examine human exposure to bats and other bushmeat in the region, ten ecologists to survey local wildlife, and four veterinarians to capture and sample bats in the index village as well as bats and other wildlife in neighboring forests (Fig1). Detailed methods are provided in the Supplementary Information.

### Permissions

We conducted this mission on behalf of the Ministère de l'Environnement et des Eaux et Forêts, and we benefited greatly from the support of local representatives in Guéckédou and Macenta. Other public institutions such as the Direction Préfectorale de la Santé of Guéckédou (DPS; the prefectural Guinean public health directorate) were extremely helpful, particularly in providing information about the index location of the outbreak. To the best of our abilities, we have communicated results and impressions back to local authorities and villagers.

### Anthropological investigations

Through a combination of informal discussions, formal interviews and direct observations with hunters, their families and bushmeat sellers, we gathered information on who in the region is involved in hunting, hunting methods as well as meat conservation and preparation methods. To identify the particular species hunted, we used pictures and description of bat vocalizations, morphology, and behavior.

### Larger mammal density and abundance

To evaluate whether a decline in wildlife abundance has occurred concurrently or shortly preceding the current EBOV outbreak, we conducted a wildlife survey using line transects (Buckland *et al*, 2005). The Ziama Biosphere Reserve and the Classified Forest of Diécké had previously been surveyed in 2010–2011 by members of our team (Fig1A). Using the same protocol as in the previous surveys, we repeated these surveys, recording all signs of large mammals. We then compared the transect sign counts between the two time periods using generalized linear models (GLM; McCullagh & Nelder, 1989; Hicks *et al*, 2014).

### Bat capture, sampling, and species identification

Bats were captured with 12-m mist nets at different heights in Meliandou (8.62243°N, 10.06462°W) and the neighboring village Kagbadou (8.64837°N, 10.05596°W) as well as Kéléma (8.30957°N, 10.69256°W) and Ziama Biosphere Reserve (8.37695°N, 9.30524°W; *N *= 169; Fig1A). Bats were euthanized according to standard protocols. Blood and tissue samples were collected, and aliquots were preserved in RNAlater, liquid nitrogen, and 10% neutral buffered formalin. Bats were initially identified based on morphology in the field; preliminary identifications were confirmed in the laboratory by sequencing a fragment of the mitochondrial cytochrome *b* gene (Supplementary Table S3; Supplementary Information, section D).

### Safety procedures

Capture and handling of animals and samples was only conducted by trained people. Personnel safety equipment for capture included strong leather gloves, goggles, and masks. Necropsies were carried out wearing an all-over-body-suit (Tyvec), FFP 3 safety mask, face shield, arm protection, and doubled gloves. All other non-disposable equipment was disinfected with potassium hypochlorite (0.5%). Nets were disinfected every night after use with potassium hypochlorite. All rubbish and carcasses were burned after sampling and residual sharps were buried.

### EBOV PCR analyses

Bat RNA extracts were tested for the presence of EBOV RNA following the protocol by Panning *et al* (2007).

### Soil DNA analyses

Tree ashes and soil samples were collected with single-use plastic spoons from the trunk and around a recently burnt tree nearby the home of the index case that was reported to have housed a large colony of bats as well as from another hollow tree in the periphery of the village inhabited by *Hipposideros cyclops*. DNA extracts were screened for mammal DNA using a PCR system amplifying a short 16S mitochondrial DNA fragment (Supplementary Table S3; Supplementary Information, section E; Taylor, 1996; Boessenkool *et al*, 2012; Calvignac-Spencer *et al*, 2013). PCR products were then indexed for multiplex sequencing on an Illumina MiSeq platform. High-quality reads were selected and assigned to vertebrate species using custom bioinformatics scripts.

## Supporting Information

Supplementary Information

Review Process File

## References

[b1] Amman BR, Carroll SA, Reed ZD, Sealy TK, Balinandi S, Swanepoel R, Kemp A, Erickson BR, Comer JA, Campbell S (2012). Seasonal pulses of Marburg virus circulation in juvenile *Rousettus aegyptiacus* bats coincide with periods of increased risk of human infection. PLoS Pathog.

[b2] Amman BR, Nyakarahuka L, McElroy AK, Dodd KA, Tara K, Sealy AJS, Shoemaker TR, Balinandi S, Atimnedi P, Kaboyo W (2014). Marburgvirus resurgence in Kitaka mine bat population after extermination attempts, Uganda. Emerg Infect Dis.

[b3] Baize S, Pannetier D, Oestereich L, Rieger T, Koivogui L, Magassouba NF, Soropogui B, Sow MS, Keïta S, De Clerck H (2014). Emergence of Zaire Ebola virus disease in Guinea—preliminary report. N Engl J Med.

[b4] Boessenkool S, Epp LS, Haile J, Bellemain E, Edwards M, Coissac E, Willerslev E, Brochmann C (2012). Blocking human contaminant DNA during PCR allows amplification of rare mammal species from sedimentary ancient DNA. Mol Ecol.

[b5] Boiro I, Lomonossov N, Sotsinski V, Constantinov O, Tkachenko E, Inapogui A, Balde C (1987). Eléments de recherches clinico-épidémiologiques et de laboratoire sur les fièvres hémorragiques en Guinée. Bulletin Soc Pathol Exot.

[b6] Buckland ST, Anderson DR, Burnham KP, Laake JL, Armitage P, Colton T (2005). Encyclopedia of Biostatistics.

[b7] Calvignac-Spencer S, Merkel K, Kutzner N, Kühl H, Boesch C, Kappeler PM, Metzger S, Schubert G, Leendertz FH (2013). Carrion fly-derived DNA as a tool for comprehensive and cost-effective assessment of mammalian biodiversity. Mol Ecol.

[b8] Calvignac-Spencer S, Schulze JM, Zickmann F, Renard BY (2014). Clock rooting further demonstrates that Guinea 2014 EBOV is a member of the Zaïre lineage. PLoS Curr Outbreaks.

[b9] Dudas G, Rambaut A (2014). A phylogenetic analysis of Guinea 2014 EBOV Ebolavirus outbreak. PLoS Curr.

[b10] Georges AJ, Leroy EM, Renaut AA, Benissan CT, Nabias RJ, Ngoc MT, Obiang PI, Lepage JPM, Bertherat EJ, Benoni DD (1999). Ebola hemorrhagic fever outbreaks in Gabon, 1994–1997: epidemiologic and health control issues. J Infect Dis.

[b11] Gire SK, Goba A, Andersen KG, Sealfon RSG, Park DJ, Kanneh L, Jalloh S, Momoh M, Fullah M, Dudas G (2014). Genomic surveillance elucidates Ebola virus origin and transmission during the 2014 outbreak. Science.

[b12] Hicks TC, Tranquilli S, Kuehl H, Campbell G, Swinkels J, Darby L, Boesch C, Hart J, Menken SBJ (2014). Absence of evidence is not evidence of absence: discovery of a large, continuous population of Pan troglodytes schweinfurthii in the Central Uele region of northern DRC. Biol Conserv.

[b13] Khan AS, Tshioko FK, Heymann DL, Le Guenno B, Nabeth P, Kerstiens B, Fleerackers Y, Kilmarx PH, Rodier GR, Nkuku O (1999). The reemergence of Ebola hemorrhagic fever, Democratic Republic of the Congo, 1995. J Infect Dis.

[b15] Leroy EM, Rouquet P, Formenty P, Souquiere S, Kilbourne A, Froment J-M, Bermejo M, Smit S, Karesh W, Swanepoel R (2004). Multiple Ebola virus transmission events and rapid decline of central African wildlife. Science.

[b16] Leroy EM, Kumulungui B, Pourrut X, Rouquet P, Hassanin A, Yaba P, Délicat A, Paweska JT, Gonzalez J-P, Swanepoel R (2005). Fruit bats as reservoirs of Ebola virus. Nature.

[b17] McCullagh P, Nelder JA (1989). Generalized Linear Models.

[b18] Mickleburgh S, Waylen K, Racey P (2009). Bats as bushmeat: a global review. Oryx.

[b20] Olival KJ, Hayman DT (2014). Filoviruses in bats: current knowledge and future directions. Viruses.

[b21] Panning M, Laue T, Ölschlager S, Eickmann M, Becker S, Raith S, Courbot M-CG, Nilsson M, Gopal R, Lundkvist A (2007). Diagnostic reverse-transcription polymerase chain reaction kit for filoviruses based on the strain collections of all European biosafety level 4 laboratories. J Infect Dis.

[b22] Pourrut X, Souris M, Towner JS, Rollin PE, Nichol ST, Gonzalez JP, Leroy E (2009). Large serological survey showing cocirculation of Ebola and Marburg viruses in Gabonese bat populations, and a high seroprevalence of both viruses in *Rousettus aegyptiacus*. BMC Infect Dis.

[b23] Schoepp RJ, Rossi CA, Khan SH, Goba A, Fair JN (2014). Undiagnosed acute viral febrile illnesses, Sierra Leone. Emerg Infect Dis.

[b24] Swanepoel R, Leman PA, Burt FJ, Zachariades NA, Braack L, Ksiazek TG, Rollin PE, Zaki SR, Peters CJ (1996). Experimental inoculation of plants and animals with Ebola virus. Emerg Infect Dis.

[b25] Taylor PG (1996). Reproducibility of ancient DNA sequences from extinct Pleistocene fauna. Mol Biol Evol.

[b26] Walsh PD, Abernethy KA, Bermejo M, Beyersk R, De Wachter P, Akou ME, Huljbregis B, Mambounga DI, Toham AK, Kilbourn AM (2003). Catastrophic ape decline in western equatorial Africa. Nature.

[b29] World Health Organization/International Study Team (1978). Ebola haemorrhagic fever in Sudan, 1976. Report of a WHO/International Study Team. Bull World Health Organ.

