# Supplementary figures and images for: Investigating the zoonotic origin of the West African Ebola epidemic
Source: EMBO Mol Med. 2014 Dec 30;7(1):17–23. doi: 10.15252/emmm.201404792 (PMC4309665; doi:10.15252/emmm.201404792)

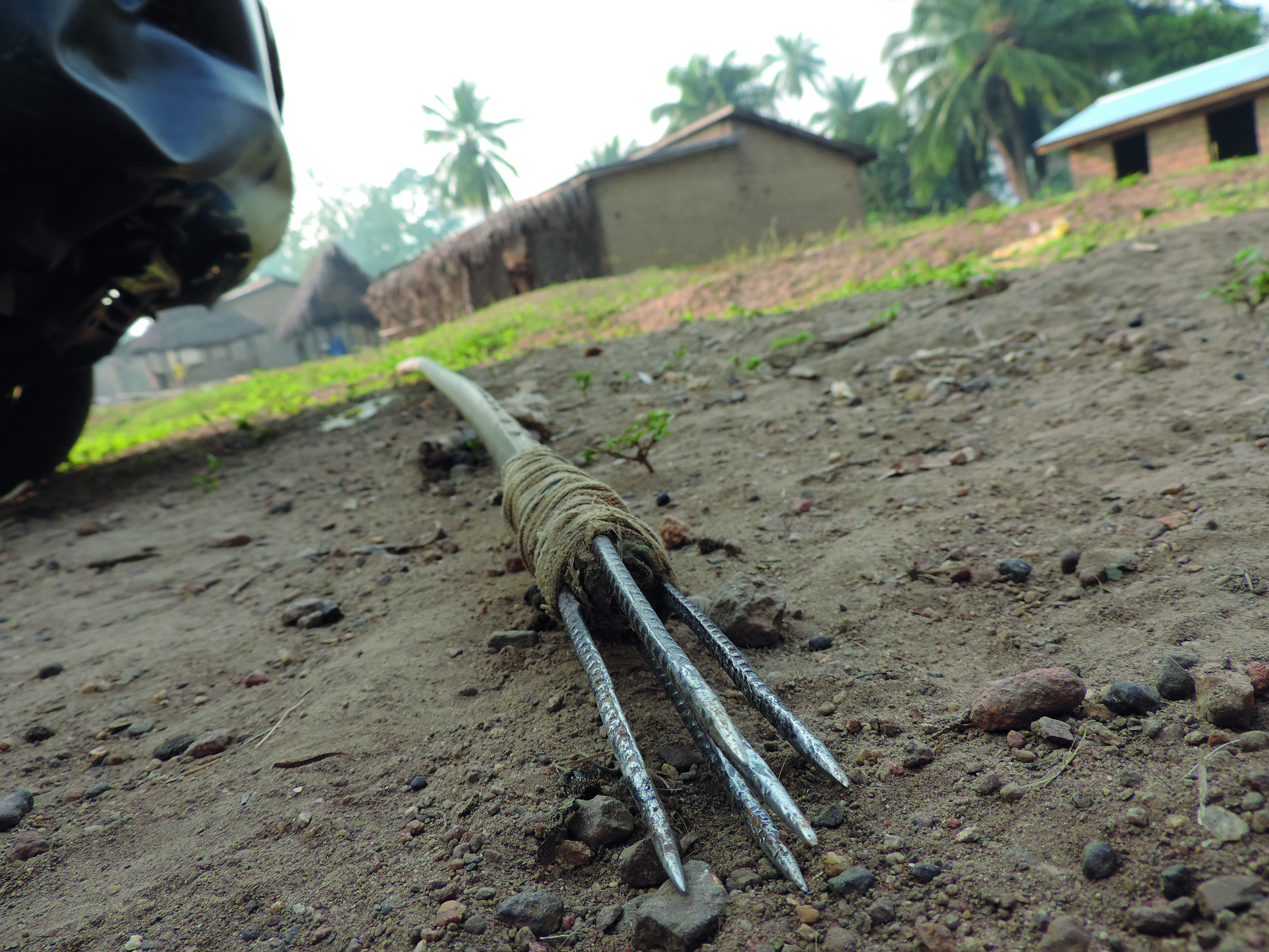

Supplement: Supplementary file 1 [file emmm0007-0017-sd1.jpg]

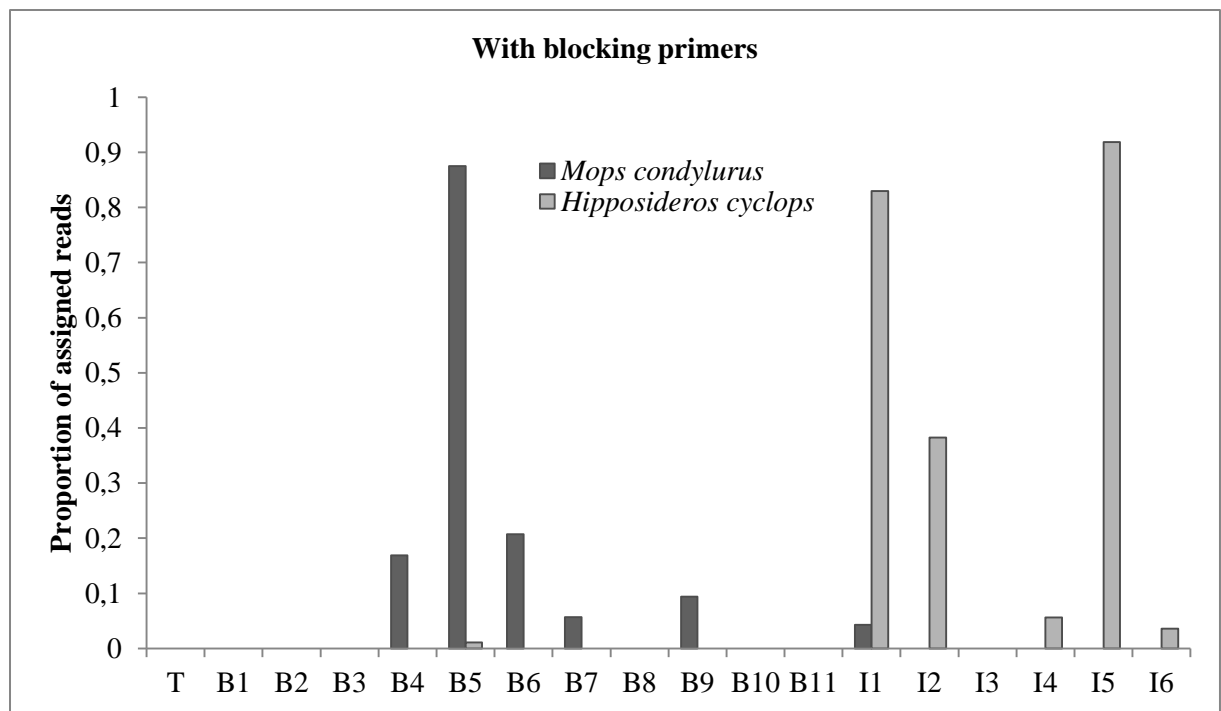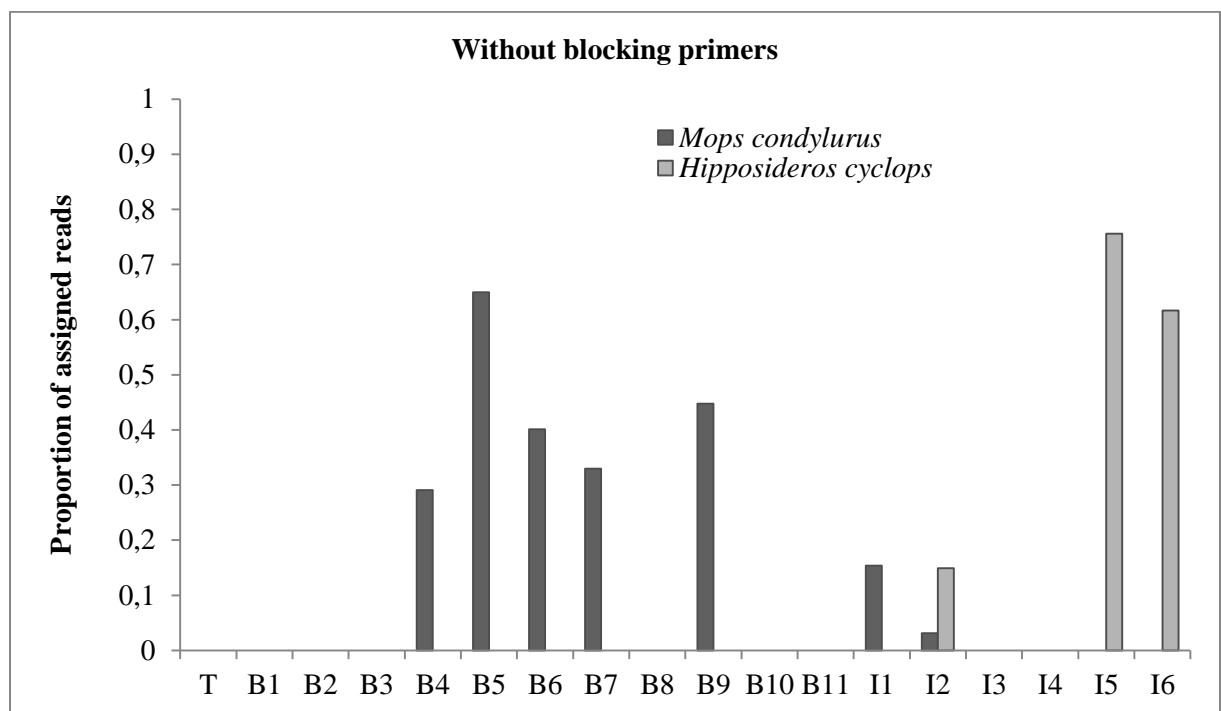

Supplement: Supplementary file 2 [file emmm0007-0017-sd2.pdf]
