# Supplementary material for: Investigating the zoonotic origin of the West African Ebola epidemic
Source: EMBO Mol Med. 2014 Dec 30;7(1):17–23. doi: 10.15252/emmm.201404792 (PMC4309665; doi:10.15252/emmm.201404792)
Supplement: Supplementary file 3 [file emmm0007-0017-sd3.docx]

**Supplementary Information. Additional descriptions and methods.**

Table of contents

[A. Description of the index village - Meliandou 2](#_Toc405973073)

[B. Anthropological investigations 3](#_Toc405973074)

[Start of the epidemic in Meliandou 4](#_Toc405973075)

[Bat hunting and preparation techniques 4](#_Toc405973076)

[Hunting and preparation techniques for other wildlife 6](#_Toc405973077)

[Domestic animals 6](#_Toc405973078)

[C. Determination of large mammal density and abundance in forest areas of the outbreak region 7](#_Toc405973079)

[D. Collection and analyses of bat samples 8](#_Toc405973080)

[Capture of bats and sample collection 8](#_Toc405973081)

[Determination of bat species 9](#_Toc405973082)

[Analyses for EBOV 10](#_Toc405973083)

[E. Collection and analyses of soil samples from the burned tree formerly housing a bat colony 10](#_Toc405973084)

[F. References 12](#_Toc405973085)

Figure legends S1 and S2 14

Table S1 14

Table S2 15

Table S3 16

# Description of the index village - Meliandou

Meliandou has been identified as outbreak index location by the DPS and the World Health Organization (Baize et al, 2014). Meliandou is situated 11km from Guéckédou and consists of 31 houses, one school and one medical center that was abandoned at the time of our visit. Most houses are made of earthen bricks and have a sheet-metal roof, outdoor kitchens and granaries, with only a few have thatched roofs (**Figure 3A**, main text). The village is surrounded by farmland: mainly rice, cacao, and coffee. Only small forest patches remain in the surrounding region. People mainly subsist on agriculture, with some trade in palm oil and vegetables (e.g., potato leaves). Before the ban of bushmeat, including bats, by the Guinean government in March 2014, people in Meliandou used to opportunistically kill and consume bats, as has been described for most regions of West Africa (Mickleburgh et al, 2009).

When our team arrived to Meliandou we first contacted the local authorities, the chief and the director of the school, to explain the aim of our mission, i.e. finding out how the epidemic may have started, and to clarify any doubts and fears. The local authorities welcomed our team and supported the mission. We hired two young men from the village to help us setting up mist nets for bat captures and let others observe us working. Best places for setting up mist nets were discussed with the villagers. These activities, as well as more in-depth contacts made by the anthropologist in our team to various people from the village, especially women, generated a friendly and trustful atmosphere. Through this good relationship we were able to gather much information, not only on hunting practices in Meliandou, but also detailed accounts of key events around the time of EBOV emergence.

# Anthropological investigations

Our anthropological aim was to study human behaviors and practices associated with hunting bats and other wildlife. We collected qualitative data in the same areas where ecologists and veterinarians were working, combining informal discussions, formal interviews and direct observation of hunters, their families and bushmeat sellers. In seven localities including Meliandou, we interviewed 14 adult males, and two adult women. In addition, in three localities including Meliandou, we opportunistically interviewed eight adult men, including one hunter, six children and youth, including four sporadic bat hunters, and four adult women including one bushmeat trader.

We asked for oral informed consent in all cases. Most interviews were conducted in French, with a minority conducted in Kissi, the local language, with the help of a translator. For interviews, we used a questionnaire asking for details of the hunting protocols, when and where individuals hunt, the tools and traps used, information on meat conservation systems used, whether bats were classified as staple food source or rarely consumed, and whether bats were sold or traded. To identify the species hunted, we used pictures and description of bats sounds, morphology, and behavior.

Domestic animals have been discussed as potential amplifying species in previous Ebola epidemics (e.g. pigs; Weingartl et al, 2013), so we also recorded all domestic animal species present while working in the index village.

### Start of the epidemic in Meliandou

According to the village’s chief, teacher and others, the index case was a two-year-old boy who died on December 28^th^, 2013. This date is several weeks later than initially reported by Baize and colleagues (Dec. 6, 2013; Baize et al, 2014). The second suspected case (2nd SC) was the three-year-old sister of the boy, who died on January 5th, 2014; again different than the death date reported by Baize and colleagues (Dec. 29, 2013; Baize et al, 2014). This date was confirmed in a condolences notebook containing the date of death and information about those present at the funeral and what presents they gave. In the same notebook, the date of death for mother, the 3rd SC, was listed as January 11th, 2014 (again differing from the death date reported by Baize and colleagues: Dec. 13, 2013; Baize et al, 2014).

Through interviews, we identified a hollow tree as a potential location for the zoonotic transmission event. Villagers reported that children played regularly in the tree and that a colony of bats lived in the tree. Unfortunately the tree was burned before we arrived in the village. The motivation for burning this tree is unclear; some villagers reported that it caught fire during an attempt to extract honey, while others suggested it was an accident that happened while children were playing with fire.

### Bat hunting and preparation techniques

Bats are classified by hunters and the local population in three categories:

*Tasa* are bats living in the surrounding farmland. According to hunters and villagers, they are big and are often found near palms or other trees looking for fruits. These descriptions likely refer to several larger fruit bat species found in the region (see below). *Tasa* are usually killed with a gun because they are found high up in palm trees. Hunters reported killing between 10 and 20 bats with a single shot. Larger bats found in caves are mainly hunted with guns. However, hunters also reported opportunisticly killing *tasa* with a long stick with nails at the end (**Supplementary** **Figure S1**) and nets. Opportunistic bat hunting is done by hitting bats with a stick or machetes and even catching them by hand. Some hunters reported selling bats when they killed large numbers; four specimens can be sold for 5,000 Guinean francs (0.7 US$). They reported burning bats to remove the hair and skin and then butchering and smoking the meat. Other hunters reported eating bats with their family and friends and not usually selling them.

*Limba* are bats living in the village inside houses and under roofs. Those bats are occasionally killed by children, who observe where the animals go in and out of the thatched roofs and use a stick or their hands to directly capture them or knock them to the ground.

*Lolibelo* is the name given to bats that are considered by villagers as flying mice rather than bats, since they smell bad, have a long tail and not much hair. *Lolibelo* are found both inside and outside of villages, under roofs and in hollow trees. These bats are also frequently caught by children using the same methods described for *limba*.

Once bats are caught, most are burned on the fire to remove their hair. Large bats hunted by adults are classically used as meat in sauces. Children often put the bats directly on a stick and grill them over a small fire. Children reported sharing these opportunistic catches with other children.

### Hunting and preparation techniques for other wildlife

According to information from hunters and authorities, traditional hunters (*kabyla donso*) are members of a family of hunters and are usually initiated by their father or uncle. They receive specific training and follow strict rules and behaviors, sometimes including taboos. Some reported hunting with the aid of a fetish and that they needed to give sacrifices to this fetish following a successful hunt. Hunters can also choose not to use a fetish, but then do not receive any supernatural powers to aid in the hunt. Christian influences made some hunters replace these sacrifices with special prayers or ablutions before hunting. Hunting is not restricted to *kabyla donso*; others may be taught to hunt by a traditional hunter and take on hunting as a profession. Besides professional hunters, many sporadic hunters exist, mostly men who opportunistically kill animals, mainly bats or rodents, while working in their fields.

### Domestic animals

We asked villagers which animals they were keeping. In Meliandou, villagers keep sheep, goats, chickens and ducks as well and a small number of cats and dogs (we counted 3 cats and 5 dogs). Not a single pig exists in the index village.

# Determination of large mammal density and abundance in forest areas of the outbreak region

To evaluate whether a decline in wildlife abundance has occurred concurrently or shortly preceding the current Ebola outbreak, we conducted a wildlife survey using standard line transect methodology (Buckland et al, 2005). Two of the three visited areas had been previously surveyed in 2010-2011 by the Wild Chimpanzee Foundation: the Ziama Biosphere Reserve and the Classified Forest Diécké. Using the same protocol as in the previous survey, we recorded all signs of large mammals, including dung, footprints, nests, sightings and vocalizations along 14 transects with a total length of 31.8km (previous survey 301.4km).

We then compared the transect sign counts for carnivores, chimpanzees, small bodied primates, duikers, other mammals and humans using generalized linear models (GLM; Hicks et al, 2014). For each response we ran a GLM with negative binomial error term with the pre-Ebola and Ebola period as a factor with two levels included. We also added an autocorrelation term to account for potential autocorrelation effects (Murai et al, 2013) and an offset term to account for different transect length (Hedley & Buckland, 2004). We did not detect any decline in mammal sign encounter rate in any of the forests where comparative data have been available. For chimpanzees and small bodied primates the positive estimates for ‘period’ and associated p-values of 0.07 and 0.01 even indicate a trend or increase in the number of signs observed during the two surveys, respectively (**Supplementary Table S1**).

# Collection and analyses of bat samples

### Capture of bats and sample collection

Bats were captured in Kéléma and Ziama Biosphere Reserve as well as Meliandou and the neighboring village Kagbadou with 2.5m x 12m mist nets (Vohwinkel, Germany) at different heights, to account for different flight heights of bat species. Nets were mainly installed at spots indicated by local people to have a high density of bats. In Kéléma one canopy net which combined three 2.5m x 12m mist nets at a top height of 12.5m (Fahr & Kalko, 2011) was installed for one night and three ground nets for two nights. In Ziama between four and five ground nets were opened per night for a total of five nights. In addition, a canopy net (same as above) was opened for two nights. In Meliandou, five to seven ground nets were opened per night for a total of six nights. These were installed near ground level within and at the periphery of the village. In Kagbadou, a canopy net (same as above) and three ground nets were installed for two nights. In addition villagers indicated hollow trees inhabited by bats, around which we also placed nets.

Nets were only opened two nights in a row in the case of success on the first night. Ineffective nets were placed in a new location on the next day. Nets were opened before sunset (between 6:30 and 7:00 pm) and checked every hour (Kunz & Parsons, 2009). Due to safety concerns related to the ongoing epidemic in the region (i.e., crowded working conditions and risk of infection), the team was not able to follow currently accepted best practices and did not check the nets throughout the night in Meliandou and Kagbadou, but was forced to only come back the following morning before sunrise to check nets and collect bats. Local assistants were appointed to make sure villagers did not touch nets or animals trapped in the nets.

We captured and sacrificed a total of 169 bats. Captured bats were individually placed into cotton bags until they were processed. Sex, age, reproductive state, fore arm length, and weight was recorded for each individual.

For anesthesia a mixture of Rompun 2% (Xylazin 20mg/ml) and Ketamin (50mg/ml) (at a ratio of 1:2 and a dose of 0.6ml/ kg) was injected intramuscular into the pectoral musculature. For euthanasia animals were bled by cardiac puncture.

Tissue samples were taken in the following order: spleen, liver, kidney, lung and intestines. Samples were immediately preserved in liquid nitrogen and/or RNAlater®. To minimize the risk of contamination the gastro intestinal tract was not opened until all other organs were sampled.

Additional tissue samples were preserved in 10% neutral buffered formalin for histopathological analyses. Samples conserved in liquid nitrogen were shipped from Guinea to Germany under special permission from both countries and immediately stored at -80°C once they arrived at the Robert Koch Institut (RKI). RNAlater samples were stored at -20°C once they arrived at the RKI.

### Determination of bat species

Individuals were tentatively identified in the field based on morphology. These preliminary identifications were later confirmed or refined in the laboratory by amplifying a ~800 bp fragment of the mitochondrial cytochrome *b* gene (all primer sequences and PCR conditions in **Supplementary Table S3**). PCR products were sequenced using Sanger’s method and chromatograms analyzed using Geneious Pro v7 (Kearse et al, 2012) . BLAST results (Altschul et al, 1990) and available biological information were combined to assign sequences to species or the lowest taxon possible. All bat sequences generated for this study are available as a FASTA file archived in DRYAD (<http://doi.org/10.5061/dryad.gq567>). In total, bats belonging to at least 13 species from 6 families were captured in southeastern Guinea. Amongst these three species were previously reported as potential reservoirs for EBOV, including two fruit bat species and an insectivorous bat species ( **Supplementary Table S3**; Olival & Hayman, 2014).

A number of other bat species are known to occur in southeastern Guinea but were not captured, including some candidate reservoir species.

### Analyses for EBOV

All bats were tested for the presence of ebolavirus RNA. For extraction of RNA, spleen samples were processed using the NucleoSpin RNA II Kit (Macherey-Nagel, Düren, Germany). PCR was performed following the protocol by Panning et al (2007). EBOV RNA was not detected.

# Collection and analyses of soil samples from the burned tree formerly housing a bat colony

Tree ashes and soil samples were collected with single use plastic spoons from the trunk (at various depths; N=7) and around the burnt tree (N=4), respectively. Soil samples were also collected around another hollow tree in the periphery of village (N=6) which was inhabited by *Hipposideros cyclops* at the time of our visit (determined with molecular methods described above). Depending on the samples, 2-7g material was available and used for nucleic acid extraction with the PowerMax^TM^ Soil DNA Isolation Kit (MO BIO Laboratories, Inc., Carlsbad, CA, USA). This kit has notably been used to demonstrate the possibility to detect vertebrate sequences from ‘dirt’ DNA (Andersen et al, 2012). Extractions took place in dedicated extraction rooms and included one blank extraction for every 9 samples (blank extracts consistently revealed negative in downstream experiments). To determine whether PCR inhibitors had been efficiently removed, DNA extracts were then analyzed using a quantitative PCR inhibition test (Calvignac‐Spencer et al, 2013). As many DNA extracts exhibited significant inhibition, all extracts were re-extracted with the GeneMATRIX Stool DNA Purification Kit (Roboklon, Berlin, Germany). Using the above mentioned PCR inhibition test, no inhibition was detected after this second extraction. DNA concentration was determined with a Nanodrop device (Thermo Scientific, Waltham, MA, USA). DNA extracts were then screened for mammalian DNA using a PCR system amplifying a short 16S mitochondrial DNA fragment (about 130bp), in presence or absence of human and pig blocking primers (**Supplementary Table S3**). All assays were seeded with 200ng DNA, or if DNA concentrations were lower than 40ng/µL, than we used 5µL of extract. To minimize the risk of contamination, AmpErase® uracil N-glycosylase (Invitrogen, Carlsbad, CA, USA) was included in all reactions (all PCR performed in the laboratory make use of dUTP). PCR products were then indexed using Nextera XT Index Primers and KAPA HiFi HotStart ReadyMix (Kapa Biosystems Inc.,Wilmington, MA, USA). Libraries were quantified with KAPA Library Quant Illumina kit, pooled equimolarly, spiked with PhiX V3 control and sequenced with V2 chemistry on an Illumina MiSeq instrument (Illumina Inc., San Diego, CA, USA). For each PCR product, a FASTQ file comprising all raw reads is archived in DRYAD (<http://doi.org/10.5061/dryad.gq567>). More than 6.8 million high-quality reads were produced from 30 amplicons (14 generated with blocking primers, 16 without). All reads were mapped to a database comprising all publicly available sequences from vertebrates as well as to a reference database of 16S sequences generated from a selection of bats captured in Guinea (these sequences are also included in the DRYAD-archived FASTA file (<http://doi.org/10.5061/dryad.gq567>). Mapping results were used to assign sequences to vertebrate species using very stringent (conservative) criteria: reads were only assigned when exhibiting 100% identity to the best-hit reference sequence and less than 98% identity to the second best-hit reference sequence. In addition, we only considered that a species was really found in a PCR product when it represented >1% of the assigned reads. PCR products derived from five samples acquired from the tree inhabited by *Hipposideros cyclops* were found to contain sequences that could be assigned to this species, validating this approach. PCR products derived from five samples acquired from the burned tree were shown to comprise sequences that could be assigned to *Mops condylurus*, thereby confirming the villagers’ family level identification (**Supplementary Figure S2**).

# References

Altschul SF, Gish W, Miller W, Myers EW, Lipman DJ (1990) Basic local alignment search tool. Journal of molecular biology 215**:** 403-410

Andersen K, Bird KL, Rasmussen M, Haile J, BREUNING‐MADSEN H, Kjaer KH, Orlando L, Gilbert MTP, Willerslev E (2012) Meta‐barcoding of ‘dirt’DNA from soil reflects vertebrate biodiversity. Molecular Ecology 21**:** 1966-1979

Baize S, Pannetier D, Oestereich L, Rieger T, Koivogui L, Magassouba NF, Soropogui B, Sow MS, Keïta S, De Clerck H (2014) Emergence of Zaire Ebola virus disease in Guinea—preliminary report. New England Journal of Medicine

Buckland ST, Anderson DR, Burnham KP, Laake JL (2005) Distance sampling: Wiley Online Library

Calvignac‐Spencer S, Merkel K, Kutzner N, Kühl H, Boesch C, Kappeler PM, Metzger S, Schubert G, Leendertz FH (2013) Carrion fly‐derived DNA as a tool for comprehensive and cost‐effective assessment of mammalian biodiversity. Molecular ecology 22**:** 915-924

Fahr J, Kalko EK (2011) Biome transitions as centres of diversity: habitat heterogeneity and diversity patterns of West African bat assemblages across spatial scales. Ecography 34**:** 177-195

Hedley S, Buckland S (2004) Spatial models for line transect sampling. JABES 9**:** 181-199

Hicks TC, Tranquilli S, Kuehl H, Campbell G, Swinkels J, Darby L, Boesch C, Hart J, Menken SBJ (2014) Absence of evidence is not evidence of absence: Discovery of a large, continuous population of Pan troglodytes schweinfurthii in the Central Uele region of northern DRC. Biological Conservation 171**:** 107-113

Kearse M, Moir R, Wilson A, Stones-Havas S, Cheung M, Sturrock S, Buxton S, Cooper A, Markowitz S, Duran C (2012) Geneious Basic: an integrated and extendable desktop software platform for the organization and analysis of sequence data. Bioinformatics 28**:** 1647-1649

Kiley PJ, Varga A, Kaplan S (1988) Physiological and structural analysis of light-harvesting mutants of Rhodobacter sphaeroides. Journal of bacteriology 170**:** 1103-1115

Kunz TH, Parsons S (2009) Ecological and Behavioral Methods for the Study of Bat. p 901. John Hopkins University Press, Baltimore.

Leroy E, Baize S, Volchkov V, Fisher-Hoch S, Georges-Courbot M, Lansoud-Soukate J, Capron M, Debre P, McCormick J, Georges A (2000) Human asymptomatic Ebola infection and strong inflammatory response. The Lancet 355**:** 2210-2215

Mickleburgh S, Waylen K, Racey P (2009) Bats as bushmeat: a global review. Oryx 43**:** 217-234

Murai M, Ruffler H, Berlemont A, Campbell G, Esono F, Agbor A, Mbomio D, Ebana A, Nze A, Kühl HS (2013) Priority Areas for Large Mammal Conservation in Equatorial Guinea. PLoS ONE 8**:** e75024

Olival KJ, Hayman DT (2014) Filoviruses in bats: current knowledge and future directions. Viruses 6**:** 1759-1788

Panning M, Laue T, Ölschlager S, Eickmann M, Becker S, Raith S, Courbot M-CG, Nilsson M, Gopal R, Lundkvist A (2007) Diagnostic reverse-transcription polymerase chain reaction kit for filoviruses based on the strain collections of all European biosafety level 4 laboratories. Journal of Infectious Diseases 196**:** S199-S204

Weber N, Fahr J (2009) Assessment of the bat fauna of Gola Forest Reserve, Sierra Leone, with recommendations for monitoring programs. p 40. Ulm: Ulm University

Weingartl HM, Nfon C, Kobinger G (2013) Review of Ebola virus infections in domestic animals. Developments in biologicals 135**:** 211-218

**Figure S1. Example of nailed stick used to hunt small animals including bats.**

**Figure S2. MiSeq sequencing of PCR products derived from ash and soil DNA.** T=trash place, B=burnt tree, I=intact tree. B1-7 and I1-2: ashes and soil sampled within the trees, B8-11 and I3-6: soil sampled around the trees. Only bat species which made up to >1% assigned read for any given PCR product are plotted.

**Table S1. GLM results for the comparison of the mammal survey data before and after/during the Ebola outbreak in Guinea.**

| **Species** | **Intercept** | **Period** | **ac-term** | **p-intercept** | **p-period** | **p-ac-term** |
| --- | --- | --- | --- | --- | --- | --- |
| Carnivore | -3.77 | 1.00 | -0.21 | 0.00 | 0.37 | 0.70 |
| Chimpanzee | -3.27 | 1.91 | 1.70 | 0.00 | 0.07 | 0.00 |
| Duiker | 0.45 | -0.39 | 0.26 | 0.00 | 0.11 | 0.00 |
| Primate | -3.26 | 1.43 | -0.24 | 0.00 | 0.01 | 0.32 |
| Other mammals | 0.35 | 0.21 | 0.24 | 0.00 | 0.33 | 0.00 |
| Human | 0.77 | 0.34 | 0.46 | 0.00 | 0.06 | 0.00 |

For each group of animal species (carnivore, chimpanzee, duiker, primate, other mammals) and human signs the estimated model coefficients and p-values are given. ‘period’ represents the two time periods of the surveys in the models and the estimated coefficients refers to the post Ebola survey. ac-term refers to the autocorrelation term.

**Table S2. Species captured in southeastern Guinea.**

| **Family** | **Species** | **Location** | **# individuals** |
| --- | --- | --- | --- |
| Hipposideridae | *Hipposideros caffer/ruber* | Kagbadou | 3 |
|  |  | Meliandou | 6 |
|  |  | Ziama | 9 |
|  | *Hipposideros cyclops* | Meliandou | 23 |
|  | *Hipposideros jonesi* | Meliandou | 1 |
|  | *Hipposideros* sp. | Meliandou | 2 |
| Molossidae | *Chaerephon pumilus* | Meliandou | 1 |
|  | *Mops condylurus** | Ziama | 1 |
| Nycteridae | *Nycteris* sp. | Meliandou | 1 |
|  |  | Ziama | 5 |
| Pteropodidae | *Eidolon helvum** | Kagbadou | 6 |
|  | *Epomops buettikoferi* | Kagbadou | 5 |
|  |  | Kéléma | 2 |
|  |  | Meliandou | 3 |
|  |  | Ziama | 7 |
|  | *Hypsignathus monstrosus** | Meliandou | 1 |
|  | *Megaloglossus azagnyi* | Meliandou | 1 |
|  |  | Ziama | 2 |
|  | *Myonycteris angolensis* | Meliandou | 23 |
|  |  | Ziama | 22 |
|  | *Myonycteris leptodon^#^* | Kagbadou | 2 |
|  |  | Kéléma | 1 |
|  |  | Meliandou | 16 |
|  |  | Ziama | 2 |
|  | *Nanonycteris veldkampii* | Kagbadou | 3 |
|  |  | Kéléma | 1 |
|  |  | Meliandou | 9 |
|  |  | Ziama | 4 |
|  | *Casinycteris ophiodon* | Ziama | 1 |
| Rhinolophidae | *Rhinolophus* sp. | Ziama | 4 |
|  |  | Meliandou | 1 |
| Vespertilionidae | *Kerivoula* sp. | Kagbadou | 1 |

* Species already shown seropositive or PCR positive for ebolaviruses. # Little collared fruit bats from Central Africa (*Myonycteris torquata*) have also previously been reported as carriers of EBOV; however, West African little collared fruit bats were recently shown to be genetically isolated from Central African populations and thus a distinct species, *Myonycteris leptodon* (formerly included in *M. torquata*; Nesi et al, 2013). Their status as a potential reservoir of EBOV is unknown.

**Table S3. Primers and PCR conditions used in this study.**

| **Target** | **Fragment length (bp)** | **Primer name** | **Sequence (5' → 3')** | **Reference** | **PCR conditions** |
| --- | --- | --- | --- | --- | --- |
| Vertebrate mitochondrial cytochrome *b* | 800 | L14724 | CGAAGCTTGATATGAAAAACCATCGTTG | (Kocher et al, 1989) | 5 min at 95°C, 40 cycles [30 s at 95 °C, 30 s at 52 °C, 45 s at 72 °C], 10 min at 72 °C |
|  |  | H15506 | AGTGGRTTRGCTGGTGTRTARTTGTC | (Kocher et al, 1989) |  |
| Mammal mitochondrial 16S | 130 | 16Smam1_f | CGGTTGGGGTGACCTCGGA | (Taylor, 1996) | 7 min at 45 °C, 10 min at 95 °C, 42 cycles [30 s at 95 °C, 30 s at 64 °C, 60 s at 72 °C], 10 min at 72 °C |
|  |  | 16Smam2_r | GCTGTTATCCCTAGGGTAACT | (Taylor, 1996) |  |
|  |  | 16Smam_blkhum3 | CGGTTGGGGCGACCTCGGAGCAGAACCC—spacerC3 | (Boessenkool et al, 2012) |  |
|  |  | 16Smam_blkpig | CGGTTGGGGTGACCTCGGAGTACAAAAAAC—spacerC3 | (Calvignac‐Spencer et al, 2013) |  |
| Mammal mitochondrial 16S | 200 | 16Smam1_f | - | - | - |
|  |  | 16Smam3_r | GATGTCCTGATCCAACATCGAG | (Calvignac‐Spencer et al, 2013) |  |
