# Supplementary material for: Investigating the zoonotic origin of the West African Ebola epidemic
Source: EMBO Mol Med. 2014 Dec 30;7(1):17–23. doi: 10.15252/emmm.201404792 (PMC4309665; doi:10.15252/emmm.201404792)
Supplement: Supplementary file 4 [file emmm0007-0017-sd4.pdf]

EMBO
Molecular Medicine

Investigating the Zoonotic Origin of the West African Ebola Epidemic

Almudena Marí Saéz, Sabrina Weiss, Kathrin Nowak, Vincent Lapeyre, Fee Zimmermann, Ariane Düx, Hjalmar S. Kühl, Moussa Kaba, Sebastien Regnaut, Kevin Merkel, Andreas Sachse, Ulla Thiesen, Lili Villányi, Christophe Boesch, Piotr W. Dabrowski, Aleksandar Radonić, Andreas Nitsche, Siv Aina J. Leendertz, Stefan Petterson, Stephan Becker, Verena Krähling, Emmanuel Couacy-Hymann, Chantal Akoua-Koffi, Natalie Weber, Lars Schaade, Jakob Fahr, Matthias Borchert, Jan F. Gogarten, Sébastien Calvignac-Spencer, Fabian H. Leendertz

Corresponding author: Fabian Leendertz, Robert Koch Institut

Review timeline:

Submission date:	27 October 2014
Editorial Decision:	25 November 2014
Accepted:	10 December 2014

Editor: Céline Carret

Transaction Report:

No Peer Review Process File is available with this article, as the authors have chosen not to make the review process public in this case.
